# Novel Polymeric Composite TPPS/s-PEEK Membranes for Low Relative Humidity PEFC

**DOI:** 10.3390/polym12061431

**Published:** 2020-06-26

**Authors:** Alessandra Carbone, Maria Angela Castriciano, Luigi Monsù Scolaro, Irene Gatto

**Affiliations:** 1 CNR-ITAE Via S. Lucia Sopra Contesse 5, 98126 Messina, Italy; alessandra.carbone@itae.cnr.it; 2 CNR-ISMN, c/o Dipartimento di Scienze Chimiche Biologiche, Farmaceutiche ed Ambientali, Università di Messina, V.le F. Stagno d’Alcontres 31, 98166 Messina, Italy; lmonsu@unime.it; 3 Dipartimento di Scienze Chimiche, Biologiche, Farmaceutiche ed Ambientali, Università di Messina, V.le F. Stagno d’Alcontres 31, 98166 Messina, Italy

**Keywords:** s-PEEK, TPPS porphyrins, J-aggregates, proton conduction, PEFC, low RH

## Abstract

Composite membranes based on different wt percentages of *meso*-tetrakis-(4-sulfonatophenyl)porphyrin (TPPS) embedded in a medium sulfonation degree (50%) sulfonated poly(etheretherketone) (s-PEEK) were investigated. The successful introduction of porphyrin into the membranes and the characterization of its different species into the membrane ionic domains were carried out by spectroscopic techniques. Moreover, the effect of TPPS arrangement was investigated in terms of water retention, proton conductivity and fuel cell performance at low relative humidity (RH). It was found that the introduction of this porphyrin induces a variation of the chemical-physical parameters, such as ion exchange capacity (IEC), water up-take (W_up_ %) λ and proton concentration ([H^+^]), attributable to the interactions that occur between the sulfonic groups of the polymer and the nitrogen sites of TPPS. The TPPS, in its J-aggregated form, actively participates in the proton conduction mechanism, also maintaining the adequate water content in more drastic conditions (80 °C and 50% RH). A maximum power density value of 462 mW cm^−2^ was obtained for the s-PEEK membrane, with a 0.77 wt % content of TPPS. This evidence suggests that the presence of J-aggregates in the proton conduction channels maintains a good hydration, even if a drastic reduction of the RH of the reactant gases occurs, preventing the membrane from a dry-out effect.

## 1. Introduction

The pressing international demand of greenhouse gases emissions reduction is implemented by the development of renewable and clean energy. In this scenario, the polymer electrolyte fuel cells (PEFCs) play a key role for energy production [1,2]. In fact, their high efficiency, energy density and environmental harmlessness, make them promising devices for clean energy generation. The key component of PEFC is a polyelectrolyte membrane based on functionalized polymer, able to transfer protons from the anode to cathode compartments. Even if Nafion^®^ is the most commonly used membrane, due to its high proton conductivity, some disadvantages, such as water uptake, methanol permeability and cost, have oriented research to the study of alternative polymers. Poly-ether-ether-ketone (PEEK) is a low cost thermoplastic, widely used in the space-flight, petroleum, chemical and medical industrial fields, due to its heat and corrosion resistant properties [3,4,5]. Moreover, a wide range of literature has shown that functionalized PEEK containing sulfonic groups is suitable for PEFC applications [6,7]. The sulfonic groups are introduced in the main chain of the polymer via a direct sulfonation reaction, and the degree of functionalization produces polymer membranes with different chemical-physical properties, such as water retention, mechanical stability and proton conductivity [8,9]. For these reasons, the sulfonation degree (DS) is generally modulated according to the final application. High DS (>60%) PEEK is used for portable applications operating at low temperature (room T) and humidity (RH ~ 50%), whereas lower or medium DS (30–50%) is used for automotive applications where the temperature spans from 60 °C to 95 °C and humidification level is in the range of 25–50%. The problems related to higher temperature and humidity are mainly due to the swelling phenomenon; on the contrary, the reduction of RH causes a drop of proton conductivity, generally water assisted, and a dry-out of the membrane. Both of them produce a drop in the mechanical properties, and consequently a reduction of electrochemical performance [10]. In order to maintain an adequate proton conductivity and preserve the mechanical properties, under low RH conditions, hygroscopic and/or proton conductor fillers are introduced into the membrane, as well as blending with other polymers, with the aim of maintaining the appropriate amount of water for proton conduction [11,12,13]. The fillers generally used for this purpose are composed of ceramic oxides or functionalized oxides, polymers with specific functional groups and organic compounds [14,15,16,17,18,19,20,21,22,23,24]. In this framework, the insertion of compounds or polymers containing nitrogenous groups, due to the ability of specific interactions/bonding with the sulfonic groups, is considered fruitful to reduce the swelling effect at high temperature and to improve the proton conductivity [25,26,27,28,29,30]. In a previous work, it was demonstrated that the insertion of *5,10,15,20-meso-*tetrakis(4-sulfonatophenyl)porphyrin (TPPS) into the high DS (>60%) membrane ionic domains shows improved performance in terms of proton conductivity and stability, with respect to the pristine polymeric matrix. In the composite s-PEEK membranes, the presence of porphyrin and the occurrence of aggregation phenomena play a key role in structuring, at nano scale, the polymeric ionic domains that are responsible for performance of the material in PEMFC devices. Furthermore, on controlling the porphyrin load, we succeeded in modulating the chemical stability, the proton conductivity and the fuel cells performance [31]. The insertion of TPPS in s-PEEK membranes takes advantage of the occurrence of supramolecular interactions between the sulfonated functional groups of the s-PEEK polymer and the diacid form of the porphyrin. It is well known in the literature that, in acidic aqueous solution, the protonation of the pyrrole nitrogen atoms present in the porphyrin core (pKa ≈ 4.9) occurs [32], with the consequent formation of a protonated species able to self-assemble. The aggregation of chromophores into organized supramolecular structures, exhibiting physicochemical properties well distinct from the isolated monomers, is important from a fundamental point of view, and suitable for possible technological applications. In this respect, a side by side arrangement (J-type) is common for different classes of aromatic compounds and, due to their peculiar structural and electronic features, these aggregates have attracted large interest for their wide potential application [33,34]. In this framework, the diacid derivative of TPPS occupies a prominent place being probably one of the most studied synthetic porphyrins. TPPS J-aggregates are characterized by a linear arrangement of the chromophores and stabilized by electrostatic interactions between the negatively charged benzenesulfonate groups and the positively charged nitrogen atoms of the pyrrole rings, as well as by hydrogen bonds and stacking interactions [35,36,37,38,39,40]. Aggregation kinetics and final supramolecular structures are strictly related to the different experimental parameters, such as the nature of the acid [41], the ionic strength [38,39], organic or inorganic scaffold [42,43,44,45,46,47,48] or the reagent mixing order protocol [49,50]. In this work, the effect of different wt percentages of TPPS in s-PEEK membranes with a medium DS (50%) has been investigated. The characterization of the different species present in the membrane was carried out by spectroscopic techniques. Moreover, the effect of TPPS arrangement in the ionic domains of s-PEEK was investigated in terms of water retention, proton conductivity and fuel cell performance at low RH. We anticipate that J-aggregated TPPS actively participates in the proton conduction mechanism, also maintaining the adequate water content in more drastic conditions (80 °C and 50% RH).

## 2. Materials and Methods

PEEK was purchased from Victrex in the form of a fine powder (450PF). Sulfuric acid (H_2_SO_4_ 96%) was purchased from Carlo Erba company. *5,10,15,20-meso-*tetrakis(4-sulfonatophenyl)porphyrin (TPPS) and dimethylacetamide solvent (DMAc 99%) were purchased from the Aldrich company.

### 2.1. Functionalization Reaction

A heterogeneous sulfonation reaction was carried out with a standardized method [14], by solubilizing 10 g of PEEK polymer in 200 mL of concentrated H_2_SO_4_ (96%). The reaction was kept for 24 h at 30 °C, to obtain a sulfonation degree of about 50%. Successively, the polymer was precipitated in a water-ice mixture and left under stirring for 24 h at room temperature. Washing steps were carried out until reaching a neutral pH and, finally, the sulfonated polymer was dried at 70 °C for 24 h and at 120 °C for 2 h.

### 2.2. Membrane Preparation

The sulfonated polymer was solubilized in dimethyl acetamide (DMAc) (10 wt %) at 90 °C, under magnetic stirring. Once complete solubilization was achieved, the re-concentration phase was started (with partial evaporation of the solvent), keeping the temperature constant, until a viscosity suitable for doctor-blade casting technique was reached. The cast film was left for 1 h at 50 °C and for 3 h at 80 °C, for a complete evaporation of the solvent. The obtained membrane was heat-treated at 120 °C for 16 h, and then an acid treatment was carried out using 1 M sulfuric acid at 50 °C for 2 h, followed by washing in water for 1 h at the same temperature. Acid treatment is important for activating proton groups for ion exchange and coordination of water molecules, as well as for purifying membranes from any residual solvent. A recast and 3 composite membranes were prepared, starting from the sulfonated polymer, containing 0.35, 0.77 and 1.5 wt % of TPPS porphyrin. TPPS was added to the polymer dispersion after dissolution in DMAc, under stirring before the re-concentration phase. For further steps, the preparation of the composite membranes followed a procedure previously reported in the literature [31]. Table 1 reports the prepared membranes.

### 2.3. Chemical-Physical Characterizations

#### 2.3.1. UV-Vis Extinction and Fluorescence Emission spectroscopies

The UV-vis extinction spectra were recorded for the three DMAc composite membranes at 25 °C on an Agilent mod. 8453 diode array spectrophotometer. The membranes were stabilized at room conditions before measuring. Fluorescence emission spectra were acquired on a Jobin Yvon-Spex Fluoromax 4 spectrofluorimeter. Emission spectra were not corrected for the absorbance of the samples.

#### 2.3.2. Thickness Measurements

The thickness of the membranes was measured with a Mitutoyo digital thickness gauge (mod. Absolute). The samples, before the measurement, were equilibrates at room temperature and humidity. The thickness was measured as an average value of at least 20 points taken on the surface of each membrane and corresponds to a value of about 45 μm.

#### 2.3.3. Ion Exchange Capacity

The ion exchange capacity (IEC, meq g^−1^) was calculated through acid base titration of the membrane, performed with an automatic titrator (Metrohm Mod. Titrino 751 GPD) as reported elsewhere [51]. The IEC of the titrated membrane is therefore given by Equation (1)

IEC = V · M/m_dry_ (1)

where V is the titrant volume (mL), M is the concentration of the titrant (mol·L^−1^) and m_dry_ is the dry mass of the sample (g).

#### 2.3.4. Water Uptake, λ Parameter and Proton Concentration

The water uptake (W_up_ %) was determined as percentage by calculating the difference in weight between the wet and the dry sample according to Equation (2)

W_up_ % = [(m_wet_ − m_dry_)/m_dry_] × 100 (2)

where m_wet_ and m_dry_ are the wet and the dry mass (g) of the membrane sample, respectively.

The dry mass was determined by weighing the sample after drying in an oven for 2 h at 80 °C under vacuum (1000 mbar); the wet mass was determined by weighing the sample after immersion in H_2_O for 24 h at 30 °C and for 2 h at 80 °C.

The λ parameter, expressed as the ratio moles H_2_O/moles-SO_3_H was calculated through the water uptake and IEC values ratio.

The proton concentration, expressed in M, is calculated as follows (Equation (3)):

[H^+^] = IEC × m_dry_/V_wet_ (3)

where V_wet_ is the wet volume, expressed in cm^3^.

#### 2.3.5. In-plane Proton Conductivity and Effective Mobility

The proton conductivity (σ, S cm^−1^) in the longitudinal direction was measured with the four probes method, using a commercial cell (Bekktech) and a potentiostat-galvanostat (AMEL mod. 2049), as reported elsewhere [14]. Proton conductivity was obtained at 30 °C and 80 °C and full humidification levels (100% RH), by applying Equation (4).

σ = 1/R · (L/W · T)(4)

where R is the electrical resistance (Ω), L = 0.425 cm the fixed distance between the two platinum electrodes, W the width (cm), and T the thickness of the sample (cm).

The activation energy was calculated by the Arrhenius law, from conductivity results carried out at 100% RH. The effective proton mobility μ (cm^2^ V^−1^ s^−1^) was calculated as follows (Equation (5)):

μ = σ/([H^+^] × F) (5)

where [H^+^] is the proton concentration and F is the Faraday constant (96,485 C mol^−1^)

#### 2.3.6. Fuel Cell Tests

Homemade electrodes were prepared by spraying the catalytic ink onto a commercial gas diffusion layer Sigracet-24BC (SGL group), following a standardized procedure [31]. A Pt loading of 0.5 mg cm^−2^ for both the anode and the cathode side was used. Membrane and electrodes were directly assembled in a single cell, tightened at 10 Nm, to obtain membrane-electrodes assemblies (MEAs).

The cell tests were performed in a 25 cm^2^ cell, connected to a commercial test station (Fuel Cell Technologies). The different membranes developed were characterized by feeding humidified H_2_ to the anode and humidified air to the cathode at the temperature of 80 °C, at the absolute gas pressure of 3.0 bar and at the values of RH of 50%, 75% and 100%. The gas flows were set at 1.5 and 2 times the stoichiometry at the working current for hydrogen and air, respectively. For each measurement, the open circuit voltage (OCV) values of the membranes were also measured. The cell resistance (R_cell_) was measured at OCV with an Agilent milliohmmeter by a static method at a frequency of 1 kHz.

## 3. Results

### 3.1. Spectroscopic Characterizations

**Figure 1:**
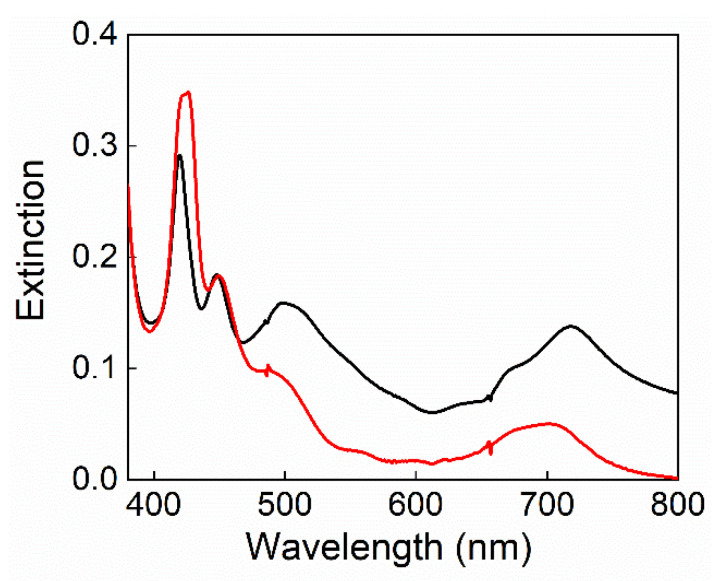
Extinction spectra of 0.77 wt % TPPS/ sulfonated poly(etheretherketone) (s-PEEK) dispersion in dimethyl acetamide (DMAc). Instantaneously after filler addition (black line) and in sol phase after 90 min at 80 °C (red line).

Taking advantage of the favorable spectroscopic properties of porphyrins, the different preparation steps and the final composite membranes with different TPPS content were characterized by UV-Vis extinction and fluorescence emission techniques. Porphyrins exhibit in the visible region (400–700 nm) a very intense Soret band with a high molar extinction coefficient (ε ≈ 10^5^ M^−1^ cm^−1^) and a pattern of weaker Q-bands at longer wavelengths (ε ≈ 10^4^ M^−1^ cm^−1^). These latter are extremely diagnostic for the determination of structural changes and/or coordination on the macrocyclic ring. A change from D_2h_ to D_4h_-symmetry leads to a simplification of the Q bands pattern with the concomitant reduction from four to two Q-bands [52]. For this class of compounds, fluorescence emission is evidenced by the presence of a typical double shaped band in the 600–800 nm range, originating from the Q (0,0) and Q (0,1) transitions. To characterize the re-casting steps, extinction spectra were recorded on the porphyrin TPPS and s-PEEK dispersion in DMAc, instantaneously after the addition of the filler and, in the sol phase (80 °C, 90 min.), before the stratification of the membranes by Doctor-Blade technique. Figure 1 shows the extinction spectra for 0.77 wt % TPPS sample taken as reference. Two distinct Soret bands centered at 420 nm and 445 nm are clearly detectable in the initial dispersion (black line), and ascribable to the porphyrin free base and its diacid form in DMAc, respectively. Moreover, a high baseline background, due to scattering of the dispersion, together with a broad band at about 500 nm are evident. According to the literature, the latter is ascribable to porphyrin J-aggregates in presence of s-PEEK polymer [31]. The thermal treatment of the sample, required for the membrane preparation, induces the solubilization of the filler, as confirmed by the decrease of baseline in the extinction spectrum (Figure 1, red line), and the substantial disappearing of the J-aggregate band. Furthermore, the Soret band of the free base TPPS evidences an increase in intensity and a bathochromic shift (Δλ = +4 nm). Therefore, high temperature treatment causes the disaggregation of preformed porphyrin aggregates and the inclusion of the porphyrin free base in the polymeric matrix, as confirmed by the red shift of the Soret band, ascribable to the location of the porphyrin in a different microenvironment, with respect to the initial sample. In accordance with the data reported in the literature, this aspect is crucial for the formation of aggregates driven by weak intermolecular forces throughout the evaporation process and for a re-organization at nanoscale of the polymeric ionic domains [31].

**Figure 2:**
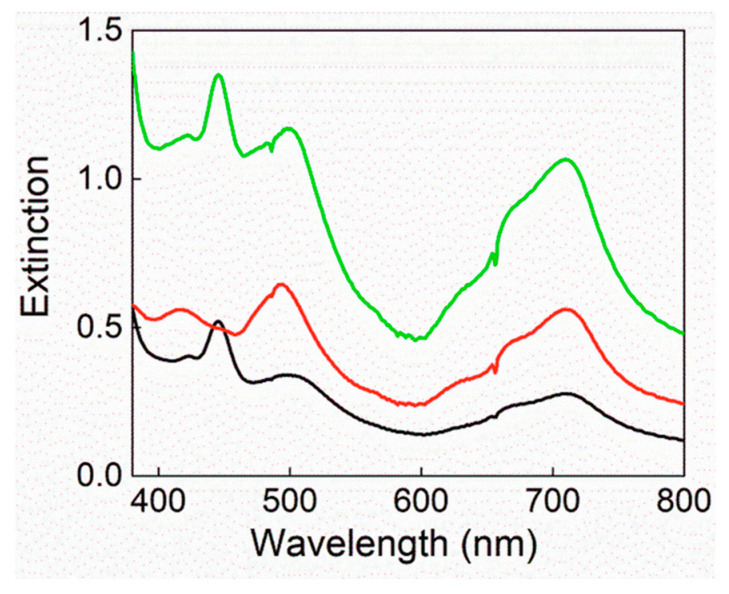
Extinction spectra of s-PEEK/TPPS composite membranes re-cast from DMAc 0.35 wt % TPPS (black line), s-PEEK-0.77 (red line) and s-PEEK-1.5 (green line) at 25 °C.

Figure 2 displays the extinction spectra of the final composite membranes obtained with different wt % TPPS. Depending on the load of TPPS, a different distribution of the porphyrin free base, its diacid form and the J-aggregate is evident from the relative bands at 422, 445 and 496 nm, respectively. In particular, for the sample s-PEEK-0.77, the extinction spectrum seems almost attributable to porphyrin J-aggregates.

**Figure 3:**
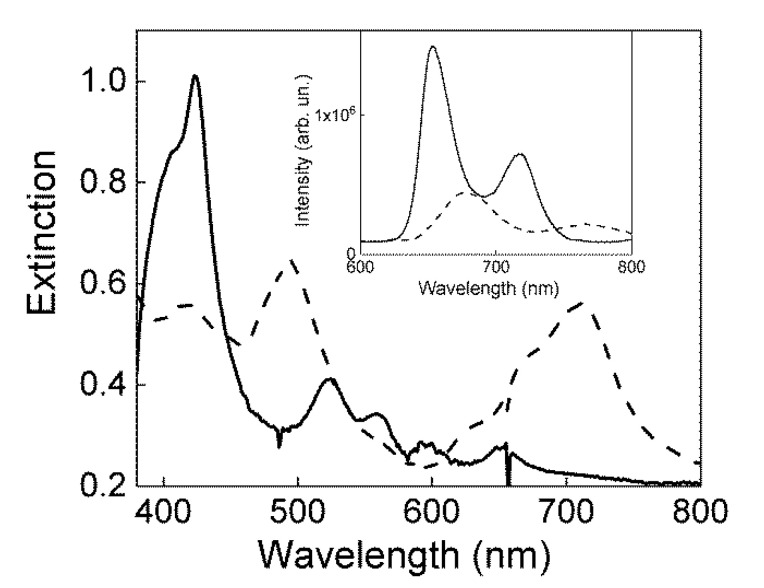
Extinction and fluorescence emission (inset) spectra of s-PEEK-0.77 composite membranes re-cast from DMAc (black dashed line) and after dipping into alkaline NaOH, 1 M solution (black solid line) at 25 °C.

Indeed, the presence of a residual amount of monomeric TPPS in s-PEEK-0.77 composite membrane is confirmed by fluorescence emission spectra. According to the literature [31], the emission spectrum (inset of Figure 3, dashed line) shows, in the range 600–800 nm, the two band profile ascribable to the diacid porphyrin embedded in the polymeric matrix, together with a contribution from J-aggregate emission. Moreover, to confirm the location of the porphyrin species into the s-PEEK ionic domains and the proton exchange process, s-PEEK-0.77 composite membrane was dipped into alkaline solution (NaOH 1 M). The corresponding extinction spectrum (Figure 3, solid black line) shows the complete disappearance of the J-aggregate band, and the exclusive presence of the Soret band centered at 424 nm, accompanied by four Q-bands at 524, 562, 598, 652, due to TPPS in its monomeric free base form. The corresponding emission spectrum (inset of Figure 3, solid black line) confirms the nature of the fluorophore embedded in the membrane. Porphyrin disaggregation in alkaline conditions has already been described for similar systems, with only a slight difference in the band positions with respect to the present system [31]. Indeed, this latter discrepancy could be due to the location of the porphyrin in a different microenvironment, due to the changed sulfonation degree for the two compared membranes.

### 3.2. IEC, Wup, λ and Proton Concentration

Chemical-physical characterizations, such as IEC, W_up_ % and λ, were carried out to understand how the different arrangements of TPPS could affect the main properties of the polymeric matrix. Regarding the IEC data, addition of TPPS to the s-PEEK is expected to increase the ion exchange capacity, due to the presence of sulfonic groups as substituents of the porphyrin structure. Moreover, it is known that interactions between the nitrogen atoms of porphyrin and the sulfonic groups of s-PEEK occur [25,31], so a reduction of ion exchange capacity is expected in the composite membranes, due to the lower availability of the exchangeable protons. Consequently, the measured IEC values should be considered as a compromise between the free proton ions, those involved in the interactions between the sulfonic groups of the polymer and TPPS and those responsible for TPPS stacking in the J-aggregates. As reported in Table 2, the introduction of TPPS increases the IEC of the s-PEEK-0.35 composite membrane respect the recast one. This behavior is attributable to the increase of the number of free protons available for ion exchange, due to the presence of the diacid porphyrin, as the predominant species into the polymer matrix. In s-PEEK-0.77, the TPPS distribution is mainly in J aggregates form (see Figure 2), and a reduction of IEC is expected with respect to 0.35 wt % load, due to the formation of a supramolecular network that involves the sulfonated groups of both the polymer and the porphyrin. An extra addition of TPPS up to 1.5 wt % causes a further reduction of IEC down to the recast value, since a higher number of SO_3_H groups of s-PEEK is involved in the hydrogen bonding with the porphyrin N-sites.

**Figure 4:**
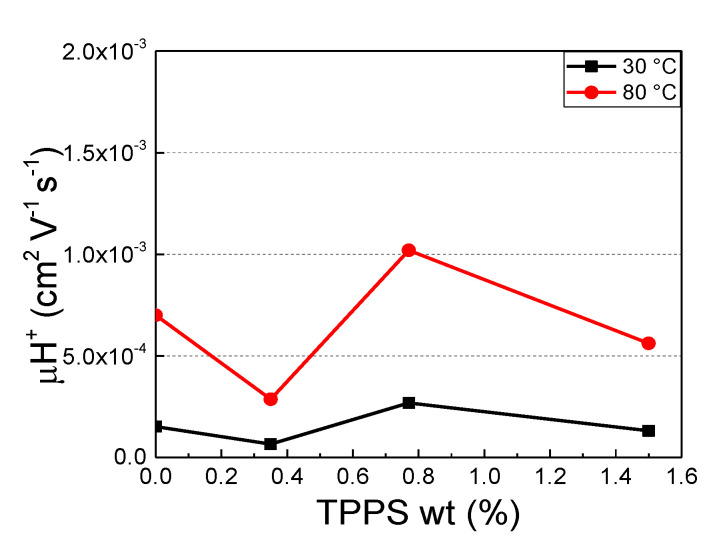
Proton mobility at different temperatures.

The ability of functional groups to retain water molecules is quantified by measuring the water uptake (W_up_ %) and calculating the λ parameter, as shown in Table 2. As expected, the W_up_ % increases by increasing temperature and, over the entire investigated temperature range, presenting a maximum value for the s-PEEK-0.77 membrane. Additionally, the λ parameter, in accordance with the W_up_ %, increases by increasing temperature and the amount of TPPS introduced. At 30 °C, the variation of λ follows the same trend as W_up_ %, and most of the water is present as loosely bound water (λ ≤ 10) [53]. At 80 °C, higher λ values are found. In particular, for the samples s-PEEK-0.77 and s-PEEK-1.5, values of 22 and 14, respectively, are obtained. This finding indicates that water is present as a second phase, which mainly contributes to the proton conduction mechanism. This effect could be correlated with the introduction of porphyrin into the membranes, thereby inducing a re-arrangement of the polymeric structure, as also revealed by SEM characterization. In fact, as reported in the Supplementary Material, Figure S1, different morphologies can be found, depending on the porphyrin loads. These results are in accordance with morphological and structural studies previously reported for similar systems [31]. Regarding the proton concentration, in this case, the data are also in agreement with the trend of water uptake and λ, presenting a maximum value for the membrane with porphyrin in J-aggregate form (s-PEEK-0.77). The proton mobility expresses the ability of the proton carriers to move into hydrated polymeric channels, and it is strictly related to the proton conductivity mechanism. From Figure 4, it is evident that the proton mobility is higher for the membrane with 0.77 wt % TPPS, than the other composite and recast membranes for both the investigated temperatures. In addition, the same trend reported for proton concentration, meaning that all the protons present in the second hydration phase (λ > 10) are involved in the conduction mechanism.

### 3.3. Electrochemical Characterizations

**Figure 5:**
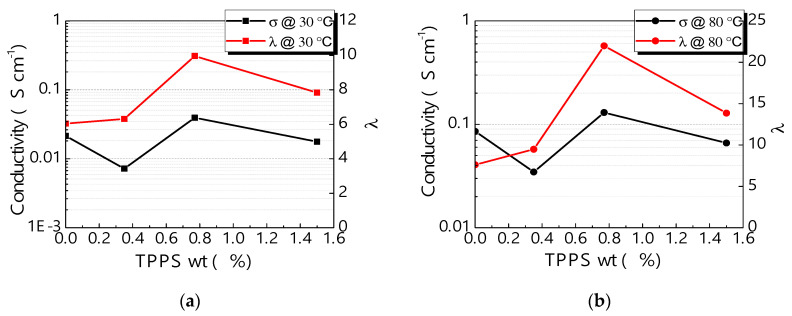
Proton conductivity and λ as a function of porphyrin loading (**a**) at 30 °C, and (**b**) at 80 °C.

Another fundamental property of polymeric membranes is the proton conductivity, closely related to the chemical-physical parameters above discussed. The proton conductivity data, as a function of TPPS loading at two different temperatures (30 °C and 80 °C) are reported in Figure 5a,b. In general, the conductivity increases by increasing the temperature from 30 to 80 °C for all the investigated samples. Moreover, for both the temperatures, a maximum in conductivity can be found for s-PEEK-0.77, in accordance with the other chemical-physical values, especially water uptake, proton concentration, mobility and λ. The data were compared to λ values to highlight the importance of the bonding among protons, water and functional groups. The same trend was found for conductivity and λ at both the temperatures, meaning that the ratio of water per sulfonic group plays a key role in the conduction mechanism.

Proton conductivity across the membrane can occur mainly by the Grotthuss and the vehicular mechanism. According to the Grotthuss mechanism, protons are transferred from one functional group to another, in a succession of hydrogen bond making and breaking; this “proton jump” determines the reorganization of the surrounding environment, usually with the orientation of the dipoles of the solvent, and therefore the formation of a proton diffusion path. According to the vehicular mechanism, the proton migration requires the translational movement of larger species, which therefore serve as diffusion vehicles. For example, in an aqueous environment, protons are transported by water molecules. The activation energy is a fundamental tool to better understand which mechanism is predominant during proton conduction. Several papers report activation energy in the range 14–40 kJ mol^−1^ for Grotthuss mechanism and lower than 14 kJ mol^−1^ for the vehicular one [54,55]. Since proton conductivity shows an Arrhenius behavior, the activation energy can be roughly calculated for the developed membranes and it is reported in Table 3.

It is evident that the conduction mechanism is mainly ruled by the Grotthuss mechanism and the lowest activation energy (21 kJ mol^−1^) was found for s-PEEK-0.77.

All the data reported so far point to the fact that porphyrins are strongly bound to the functional groups of the polymer, and their amount and reorganization in the polymeric matrix directly affect the proton conduction mechanism. Furthermore, J-aggregates seem to be actively involved in the proton conduction mechanism [31].

**Figure 6:**
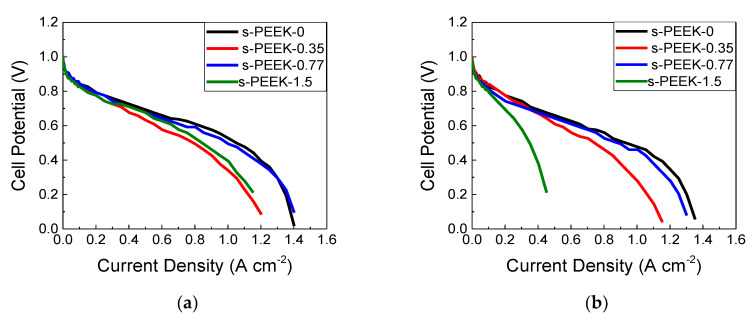
Polarization curves at 80 °C and different relative humidity (RH) levels: (**a**) 100% and (**b**) 75%.

All the characterizations above described give us indications on the real applicability of the membranes in a PEFC device. To evaluate their performance and make a comparison among the different porphyrin loaded membranes, the electrochemical characterization in terms of I-V curves was carried out. In Figure 6a the polarization curves of the four membranes obtained at 80 °C and 100% RH are compared. It is evident that s-PEEK-0.77 has the best performance among the investigated composite membranes, in accordance with the chemical-physical parameters, such as proton concentration and mobility, λ and conductivity values. Furthermore, its performance is comparable to the s-PEEK-0 recast membrane. As reported in Table 4, all the investigated samples showed a good OCV value, of about 1 V, comparable or slightly higher than that obtained from s-PEEK recast membrane. This means that the introduction of TPPS increases the OCV value indicating a more dense and compact structural arrangement of the composite membranes, with respect to that obtained by the pristine polymer. In general, the cell resistance of composite membranes is higher than recast one and among the composites the 0.77 wt % amount produces a minor increase (Table 4). In addition, the current density recorded at 0.6 V, typically referred to the ohmic region, highlights that the TPPS load plays a key role in the electrochemical performance.

**Figure 7:**
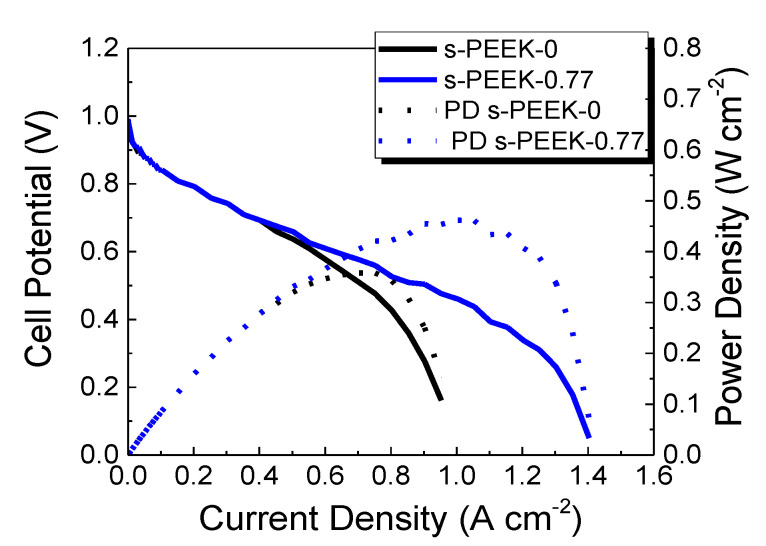
I-V and power density curves at 80 °C and 50% RH.

In Figure 6b, the effect of the decrease of relative humidity is shown, also highlighting in this case a contribution due to the porphyrin in the structure. In fact, at 100% RH, when the polymer is completely hydrated, the presence of TPPS into the polymeric matrix affects less the proton conduction mechanism. On the contrary, at lower RH, the different porphyrin load and their peculiar arrangement in J-aggregates participate within the membrane conduction channels. As expected, the reduction of humidity level down to 75% RH produces a decrease of the performance and an increase of the cell resistance for all the investigated membranes (Table 4). In particular, s-PEEK-0.77 sample provided the best performance among the three composite membranes, and was comparable to the recast one. As discussed above, this fact could be explained by the participation of porphyrins in the proton conduction mechanism, by making the membrane more hydrated, as can be seen from the data of W_up_% and λ. This result is in line with data already reported for similar systems. Indeed, previous investigations demonstrated that the physical-chemical properties of TPPS 0.77 wt % sample are related to a good balance between porphyrins in their monomeric and aggregated species. In particular, the extra sulfonated groups of the porphyrin free base enhance the proton membrane conductivity, while an improvement of the mechanical properties is ascribed to the polymer structural re-arrangement, due to the presence of the aggregates [31]. The contribution of TPPS to membrane hydration is still visible when the operative conditions were shifted toward low RH (50%) Figure 7 shows a comparison of the I-V and power density curves for the s-PEEK-0 and s-PEEK-0.77 membranes. For samples containing 0.35 and 1.5 wt % of TPPS, the polarization curves were not recorded, due to poor performance in such drastic conditions. The curves evidence that the reduction of gas humidification affects the electrochemical performance. Indeed, the performance of the s-PEEK-0.77 membrane remains quite unaltered with respect to the 75% RH ones, and it is significantly higher than that of the recast. This effect is due to the different hydration of the membrane, as a consequence of the presence of porphyrin. In particular, the J-aggregate in the composite membrane not only participates in the proton conduction, but also helps to maintain a higher level of hydration. On the contrary, a reduction of RH from 75% down to 50% drastically reduces the performance of the recast membrane, due to the dehydration of the polymer.

The power density curves indicate that s-PEEK-0.77 composite membrane reaches a maximum value of 462 mW cm^−2^, against a value of 358 mW cm^−2^ obtained with the s-PEEK-0 sample. It is notable how the presence of J-aggregates in the proton conduction channels keeps the membrane hydrated, preventing the membrane from dry-out effect, even if a drastic reduction of the RH of the reactant gases occurs.

## 4. Conclusions

Ion exchange composite membranes based on polyetheretherketone sulfonate (s-PEEK) with a medium DS (50%) and containing different wt percentages (0–1.5 wt %) of porphyrin (TPPS) as a filler have been developed for use as components in polymer electrolyte fuel cells. The successful introduction of the organic macrocycle into the membranes and the characterization of the different species were carried out by spectroscopic techniques.

A complete chemical-physical and electrochemical characterization was performed in order to investigate the effect of TPPS on the properties of the membranes. It was found that the TPPS, in its J-aggregate form, actively participates in the proton conduction mechanism maintaining the adequate water content, even in more drastic conditions of 80 °C and 50% RH. In fact, the introduction of this porphyrin induces a variation of the chemical-physical parameters such as IEC, W_up_ %, λ and [H^+^], attributable to the interactions that occur between the sulfonic groups of the polymer and the nitrogen atoms of TPPS.

A maximum in proton conductivity can be found for membrane with 0.77 wt % TPPS loading, in accordance with the other chemical physical characterizations. Moreover, the lowest activation energy (21 kJ mol^−1^) found for this sample points to a conduction mechanism mainly ruled by the Grotthuss model.

The electrochemical characterization in terms of I-V curves highlights the fact that s-PEEK-0.77 has the best performance among the investigated composite membranes, especially when operating in a more drastic conditions (i.e., 50% RH). A maximum power density value of 462 mW cm^−2^ for the s-PEEK-0.77 was obtained. This finding points to the active role of J-aggregates in the proton conduction channels, keeping the membrane hydrated and preventing the membrane from dry-out effect, even if a drastic reduction of the RH of the reactant gases occurs.

## Figures and Tables

**Table 1:** Identification labels for membranes under investigation and relative *5,10,15,20-meso-*tetrakis(4-sulfonatophenyl)porphyrin (TPPS) content.

Membranes	TPPS Loading, %
s-PEEK-0	0
s-PEEK-0.35	0.35
s-PEEK-0.77	0.77
s-PEEK-1.5	1.5

**Table 2:** Ion exchange capacity (IEC) W_up_ %, λ and [H^+^] of the four membranes at different temperatures.

Temperature	Samples	IEC, meq g^−1^	W_up_, %	λ, mol (H_2_O/SO_3_H)	[H^+^], M
30 °C	s-PEEK-0	1.49	16.2	6	1.43
	s-PEEK-0.35	1.77	20.1	6	1.12
	s-PEEK-0.77	1.69	30.3	10	1.52
	s-PEEK-1.5	1.46	20.6	8	1.39
80 °C	s-PEEK-0	-	20.4	8	1.26
	s-PEEK-0.35	-	30.2	9	1.25
	s-PEEK-0.77	-	66.9	22	1.32
	s-PEEK-1.5	-	36.4	14	1.22

**Table 3:** Activation energy for the developed membranes.

Samples	E_att_/kJ mol^−1^
s-PEEK-0	31
s-PEEK-0.35	38
s-PEEK-0.77	21
s-PEEK-1.5	32

**Table 4:** Electrochemical parameters for the membranes under investigation.

	80 °C 100% RH			80 °C 75% RH			80 °C 50% RH		
Samples	OCV, V	R, Ωcm^2^	C.D. @0.6 V Acm^−2^,	OCV, V	R, Ωcm^2^	C.D. @0.6 V Acm^−2^,	OCV, V	R, Ωcm^2^	C.D. @0.6 V Acm^−2^,
s-PEEK-0	0.960	0.184	0.854	0.941	0.207	0.650	0.958	0.325	0.552
s-PEEK-0.35	1.000	0.258	0.550	1.000	0.425	0.500	-	-	-
s-PEEK-0.77	0.991	0.214	0.803	0.991	0.238	0.650	0.991	0.245	0.602
s-PEEK-1.5	1.007	0.275	0.650	0.991	0.500	0.275	-	-	-

## Supplementary Materials

The following are available online at https://www.mdpi.com/2073-4360/12/6/1431/s1, Figure S1: SEM Images of s-PEEK composite membranes, (a) s-PEEK; (b) s-PEEK-0.35; (c) s-PEEK-0.77; (d) s-PEEK-1.5; Figure S2: Extinction spectra of s-PEEK-0.77 composite membranes sulphonation degree 35% re-cast from DMAc (red line) and after dipping into alkaline NaOH, 1 M solution (black line) at 25 °C.
