# Supplementary material for: Novel Polymeric Composite TPPS/s-PEEK Membranes for Low Relative Humidity PEFC
Source: Polymers (Basel). 2020 Jun 26;12(6):1431. doi: 10.3390/polym12061431 (PMC7361698; doi:10.3390/polym12061431)
Supplement: Supplementary file 1 [file polymers-12-01431-s001.pdf]

Electronic Supporting Information

For

Novel Polymeric Composite TPPS/s-PEEK Membranes for Low Relative Humidity PEFC

Alessandra Carbone[¤], Maria Angela Castriciano[†*], Luigi Monsù Scolaro.[# †], Irene Gatto[¤*]

[¤] CNR-ITAE Via S. Lucia sopra Contesse, 5 98126, Messina, Italy.
[†] CNR-ISMN, c/o Dipartimento di Scienze Chimiche Biologiche, Farmaceutiche ed Ambientali, Università di Messina, V.le F. Stagno d'Alcontres 31, 98166 Messina.
[#] Dipartimento di Scienze Chimiche, Biologiche, Farmaceutiche ed Ambientali, Università di Messina, V.le F. Stagno d'Alcontres 31, 98166 Messina.
[*] Correspondence: irene.gatto@itae.cnr.it; Tel.: +39-090624231 (I.G.)
maria.castriciano@cnr.it; (M.C.)

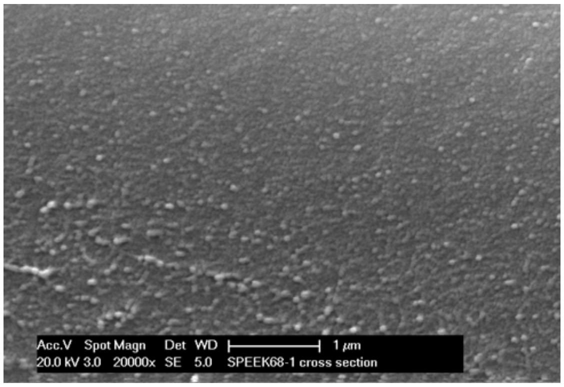

a)

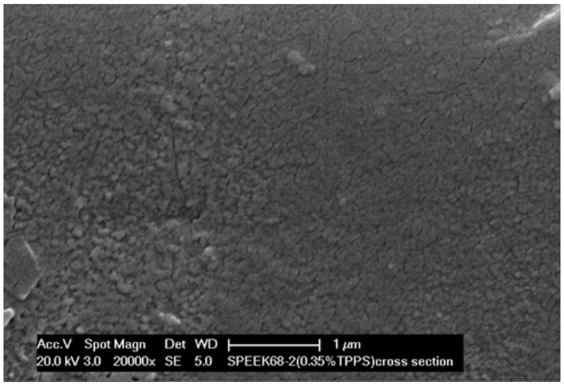

b)

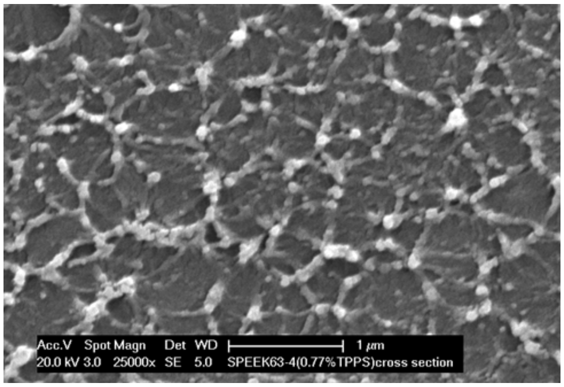

c)

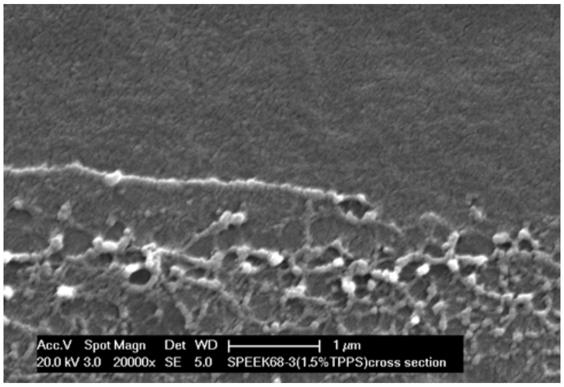

d)

Figure S1. SEM Images of s-PEEK composite membranes, a) s-PEEK; b) s-PEEK-0.35; c) s-PEEK-0.77; d) s-PEEK-1.5.

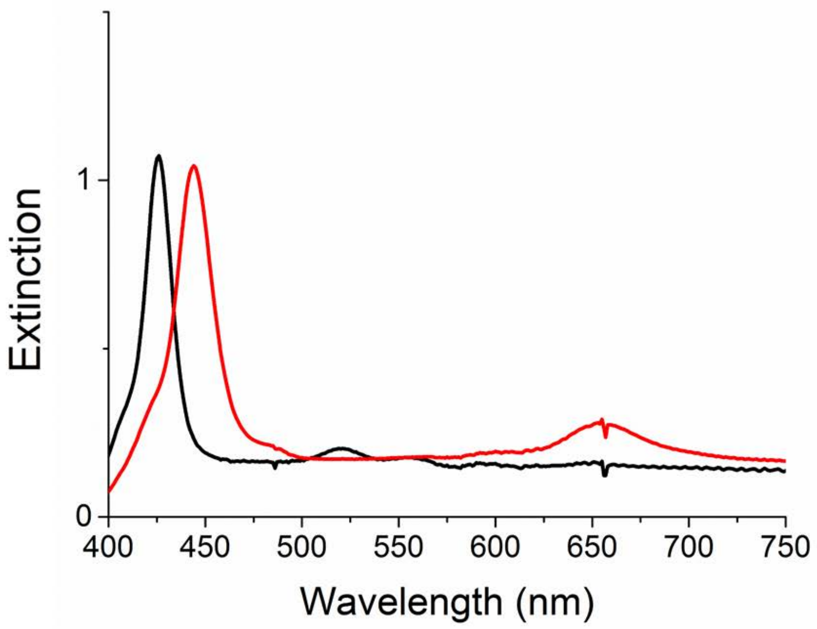

Figure S2. Extinction spectra of s-PEEK-0.77 composite membranes sulphonation degree 35% re-cast from DMAc (red line) and after dipping into alkaline NaOH, 1 M solution (black line) at 25 ° C.
